# Supplementary material for: Impaired vitamin D signaling reveals neutrophils as key drivers of prostate cancer dissemination
Source: EMBO Mol Med. 2026 Apr 10;18(5):1967–89. doi: 10.1038/s44321-026-00417-5 (PMC13179334; doi:10.1038/s44321-026-00417-5)
Supplement: Supplementary file 1 — Table EV1 [file 44321_2026_417_MOESM1_ESM.zip › Table_EV1.docx]

**Table EV1 :** List of genes shared between DEGs in luminal DLVP cells of *Pten/Vdr^(i)pe-/-^* compared to *Pten^(i)pe-/-^* mice and in Luminal C cluster identified by scRNA-seq.
