# Supplementary material for: Impaired vitamin D signaling reveals neutrophils as key drivers of prostate cancer dissemination
Source: EMBO Mol Med. 2026 Apr 10;18(5):1967–89. doi: 10.1038/s44321-026-00417-5 (PMC13179334; doi:10.1038/s44321-026-00417-5)
Supplement: Supplementary file 2 — Table EV2 [file 44321_2026_417_MOESM2_ESM.zip › Table_EV2.docx]

**Table EV2 :** Output file from the ClusterProfiler analysis using differentially expressed genes from *Pten/Vdr^(i)pe-/-^* and *Pten^(i)pe-/-^* mice, 3 months after gene inactivation (Dataset EV6). ID and Description correspond to the name of the KEGG pathway. SetSize is the number of gene within the pathway. Enrichment Score (ES), Normalised ES, pvalue, p.adjust, qvalue, rank, and leading_edge are the statistical values of the gene set enrichment analysis. Core_enrichment depicts the differentially expressed genes within the corresponding pathway. Path confirms that we used the KEGG database. Orig indicates the order of the analysis: *Pten/Vdr^(i)pe-/-^* (V3) vs *Pten^(i)pe-/-^* (P3) mice, 3 months AGI.
