# Supplementary material for: Impaired vitamin D signaling reveals neutrophils as key drivers of prostate cancer dissemination
Source: EMBO Mol Med. 2026 Apr 10;18(5):1967–89. doi: 10.1038/s44321-026-00417-5 (PMC13179334; doi:10.1038/s44321-026-00417-5)
Supplement: Supplementary file 3 — Appendix [file 44321_2026_417_MOESM3_ESM.pdf]

Impaired vitamin D signaling reveals neutrophils as key drivers of prostate cancer dissemination

Kateryna Len-Tayon et al.

Table of content :

Appendix Fig. S1 2
Appendix Fig. S2. 3
Appendix Fig. S3. 4
Appendix Fig. S4. 5

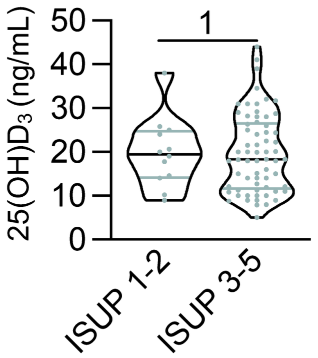

Appendix Fig. S1. Circulating 25(OH)D3 levels in patients with ISUP 1-2 or ISUP 3-5 grade PCa. The p value determined by Student's t-test is indicated.

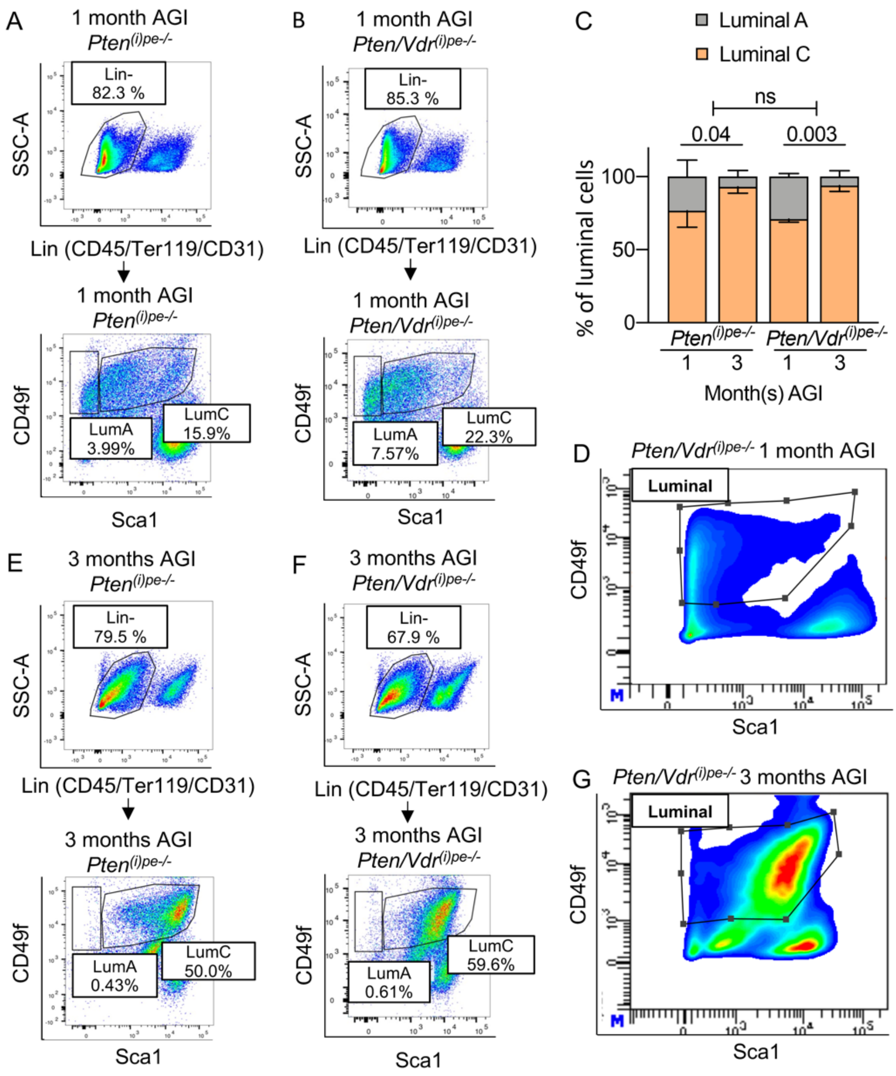

Appendix Fig. S2. Characteristics of FACS-isolated luminal cells used for transcriptome analysis. Representative gates for flow cytometry analysis of luminal cell populations from dissociated DLVPs of Pten$^{(i)pe-/-}$ 1 month (A), Pten/Vdr$^{(i)pe-/-}$ 1 month (B), Pten$^{(i)pe-/-}$ 3 months (E) and Pten/Vdr$^{(i)pe-/-}$ 3 months (F) AGI. n = 3 mice/group. C. Flow cytometry analysis of alive (DAPI-) luminal A (Lin-/CD49f+/Sca1-) and luminal C (Lin-/CD49f+/Sca1+) cells from dissociated DLVPs of Pten$^{(i)pe-/-}$ and Pten/Vdr$^{(i)pe-/-}$ mice 1 and 3 months AGI. n = 3 mice/group. p values determined by two-way ANOVA with Tukey's post-hoc are indicated. ns, $p \geq 0.05$. Data are represented as mean + or - standard deviation. Representative gates of FACS-isolated luminal cells (Lin-/CD49f+/Sca1- and Sca1+) used for RNA-seq analysis from DLVP of Pten/Vdr$^{(i)pe-/-}$ 1 month (D) and 3 months (G) AGI. n = 3/group.

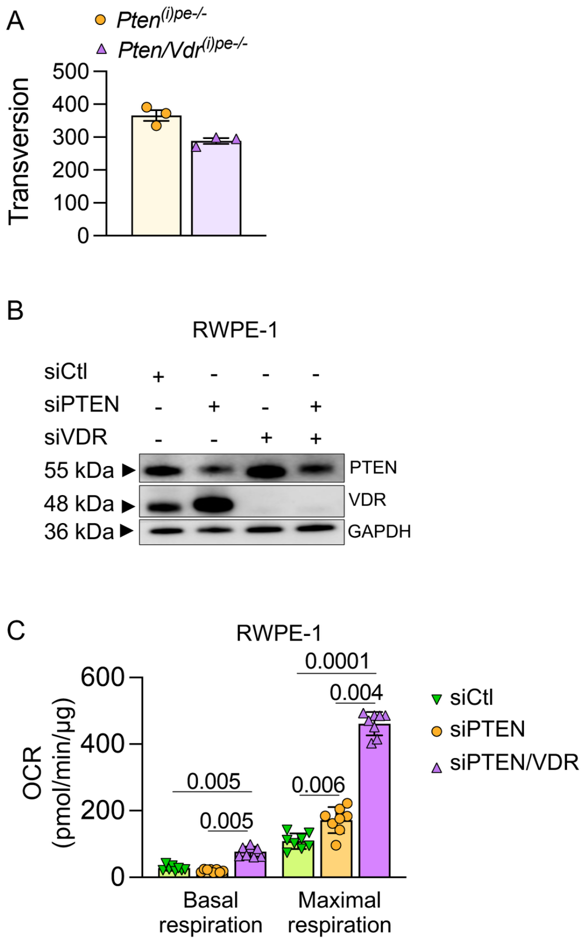

Appendix Fig. S3. **Transversion in PECs and mitochondrial respiration in VDR and/or PTEN-silenced RWPE-1 cells. A**. Number of transversion in the RNAseq datasets shown in Fig 2A. Data are represented as mean +/- standard deviation. **B**. Uncropped immunoblot analysis of PTEN and VDR in RWPE-1 cells transfected with non-targeting control (siCtrl), PTEN (siPTEN), and/or VDR (siVDR) siRNAs for 48 h. GAPDH was used as a loading control. **C**. Relative quantification of basal and maximal respiration determined with a Seahorse extracellular flux analyzer in RWPE-1 cells transfected as indicated. n = 8 per condition. Data are represented as mean +/- standard deviation. p value determined by two-way ANOVA is indicated.

	cell number	
**cluster**	*Pten*$^{(i)pe-/-}$	*Pten/Vdr*$^{(i)pe-/-}$
**Neutrophil**	1591	939
**Stroma**	1483	2848
**Tcells**	703	630
**Leukocytes**	675	1038
**Luminal**	999	936
**Endo**	113	172
**Basal**	153	116
**Bcells**	93	87

Appendix Fig. S4. Cell number in the cellular clusters identified in the scRNAseq dataset.
