# Supplementary material for: Impaired vitamin D signaling reveals neutrophils as key drivers of prostate cancer dissemination
Source: EMBO Mol Med. 2026 Apr 10;18(5):1967–89. doi: 10.1038/s44321-026-00417-5 (PMC13179334; doi:10.1038/s44321-026-00417-5)
Supplement: Supplementary file 5 — Dataset EV1 [file 44321_2026_417_MOESM5_ESM.zip › Dataset_EV1.docx]

**Dataset EV1 :** Output file from the DESeq2 analysis using the RNAseq analysis of FACS-isolated luminal cells of the DLVP from *Pten/Vdr^(i)pe-/-^* and *Pten^(i)pe-/-^* mice, 1 month after gene inactivation. BaseMean is the average of the normalized count values, dividing by size factors, taken over all samples. Log2FoldChange corresponds to the differential expression, with lfcSE the standard error. Stat is the value of the test statistic for the gene or transcript. P-value of the test for the gene or transcript and Adjusted P-value (padj) for multiple testing for the gene or transcript. NCBI and GeneName result for transcript ID and Ensembl name, respectively.
