# Supplementary material for: Impaired vitamin D signaling reveals neutrophils as key drivers of prostate cancer dissemination
Source: EMBO Mol Med. 2026 Apr 10;18(5):1967–89. doi: 10.1038/s44321-026-00417-5 (PMC13179334; doi:10.1038/s44321-026-00417-5)
Supplement: Supplementary file 7 — Dataset EV3 [file 44321_2026_417_MOESM7_ESM.zip › Dataset_EV3.docx]

**Dataset EV3 :** Output file obtained by the *FindConservedMarkers* function from Seurat to get the signatures from each cluster in the prostates from *Pten/VDR^(i)pe-/-^ and Pten^(i)pe-/-^ mice*. Values from B-F are from *Pten^(i)pe-/-^* mice, and those from G-K from *Pten/VDR^(i)pe-/-^* mice*.* p_val : p-value (unadjusted) ; avg_log2FC : log 2 fold-change of the average expression between the two groups. Positive values indicate that the feature is more highly expressed in the indicated cluster (column P); pct.1 : The percentage of cells where the feature is detected in the indicated cluster (column P); pct.2 : The percentage of cells where the feature is detected in the other clusters*.* p_val_adj : Adjusted p-value, based on Bonferroni correction using all features in the dataset. max_pval and min_pval : maximum and minimum p-values of the two samples (note: not looking at the adjusted p-values), respectively.
