# Supplementary material for: Impaired vitamin D signaling reveals neutrophils as key drivers of prostate cancer dissemination
Source: EMBO Mol Med. 2026 Apr 10;18(5):1967–89. doi: 10.1038/s44321-026-00417-5 (PMC13179334; doi:10.1038/s44321-026-00417-5)
Supplement: Supplementary file 8 — Dataset EV4 [file 44321_2026_417_MOESM8_ESM.zip › Dataset_EV4.docx]

**Dataset EV4 :** Output file obtained by the *FindMarkers* function from Seurat to get the differentially expressed gene in each cluster between the prostates from *Pten/VDR^(i)pe-/-^ and Pten^(i)pe-/-^ mice*. p_val : p-value (unadjusted) ; avg_log2FC : log 2 fold-change of the average expression between the two groups. Positive values indicate that the feature is more highly expressed in *Pten/VDR^(i)pe-/-^ mice*; pct.1 : The percentage of cells where the feature is detected in *Pten/VDR^(i)pe-/-^ mice*; pct.2 : The percentage of cells where the feature is detected in *Pten^(i)pe-/-^ mice*; p_val_adj : Adjusted p-value, based on Bonferroni correction using all features in the dataset.
