# Supplementary material for: Impaired vitamin D signaling reveals neutrophils as key drivers of prostate cancer dissemination
Source: EMBO Mol Med. 2026 Apr 10;18(5):1967–89. doi: 10.1038/s44321-026-00417-5 (PMC13179334; doi:10.1038/s44321-026-00417-5)
Supplement: Supplementary file 9 — Dataset EV5 [file 44321_2026_417_MOESM9_ESM.zip › Dataset_EV5.docx]

**Dataset EV5 :** Output file from the ClusterProfiler analysis using differentially expressed genes from the single cell analysis from *Pten/Vdr^(i)pe-/-^* and *Pten^(i)pe-/-^* mice in each cluster (Dataset EV4). ID and Description correspond to the name of the KEGG pathway. GeneRatio is the number of gene within the pathway / total number of gene in this pathway. BgRatio : number of gene in this pathway / total number of gene in the dataset used. Pvalue and qvalue, are the statistical values of the gene set enrichment analysis. geneID depicts the differentially expressed genes within the corresponding pathway. Count is the number of gene associated with this pathway in the dataset.
