# Supplementary figures and images for: Impaired vitamin D signaling reveals neutrophils as key drivers of prostate cancer dissemination
Source: EMBO Mol Med. 2026 Apr 10;18(5):1967–89. doi: 10.1038/s44321-026-00417-5 (PMC13179334; doi:10.1038/s44321-026-00417-5)

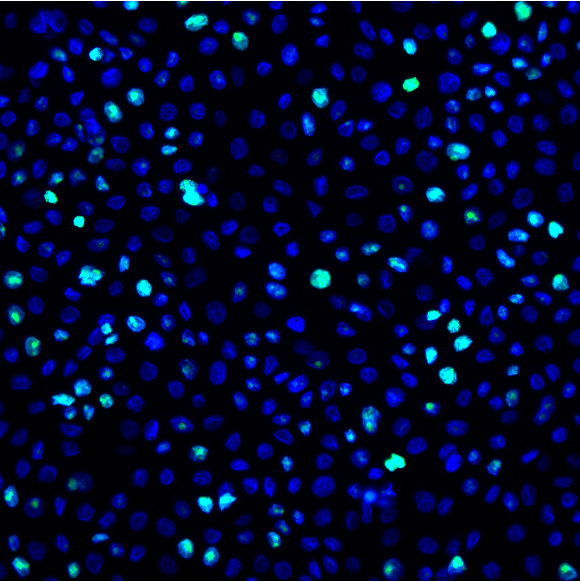

Supplement: Supplementary file 12 — Source data Fig. 2 [file 44321_2026_417_MOESM12_ESM.zip › Figure 2/2M/NAC_SiPTEN.png]

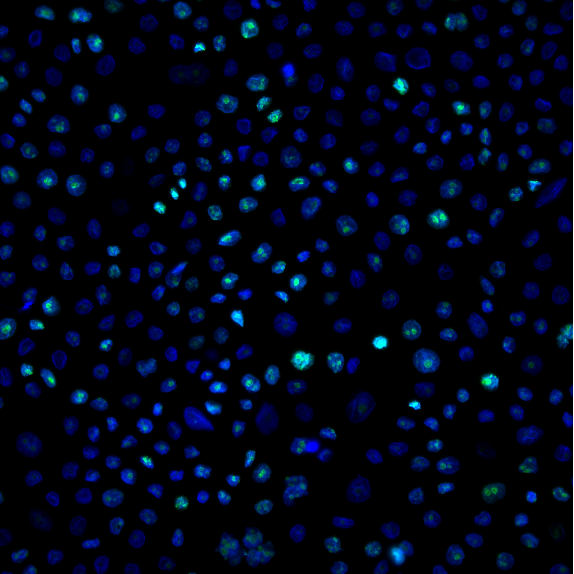

Supplement: Supplementary file 12 — Source data Fig. 2 [file 44321_2026_417_MOESM12_ESM.zip › Figure 2/2M/SiCtrl.png]

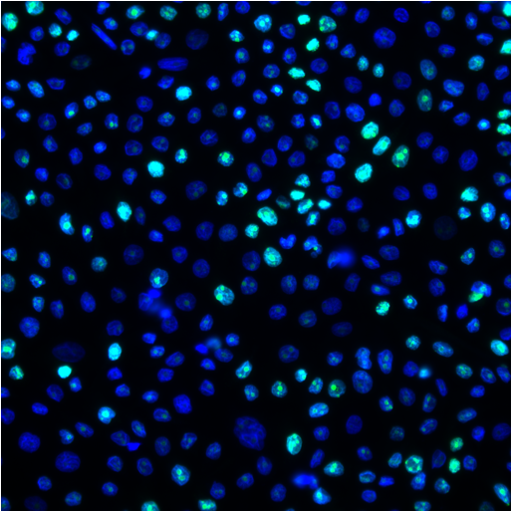

Supplement: Supplementary file 12 — Source data Fig. 2 [file 44321_2026_417_MOESM12_ESM.zip › Figure 2/2M/NAC_SiCtrl.png]

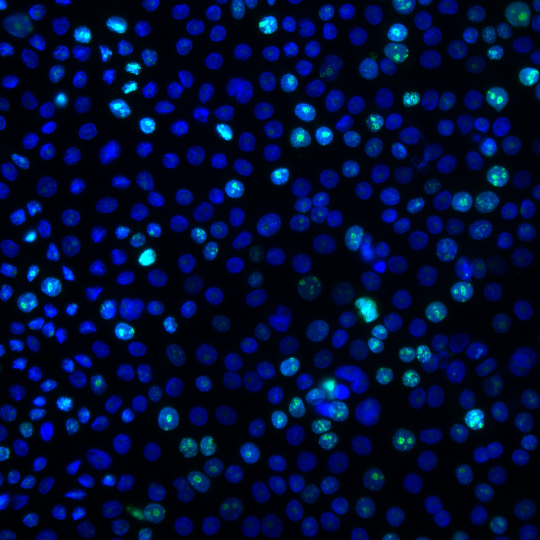

Supplement: Supplementary file 12 — Source data Fig. 2 [file 44321_2026_417_MOESM12_ESM.zip › Figure 2/2M/NAC_SiPTENVDR.png]

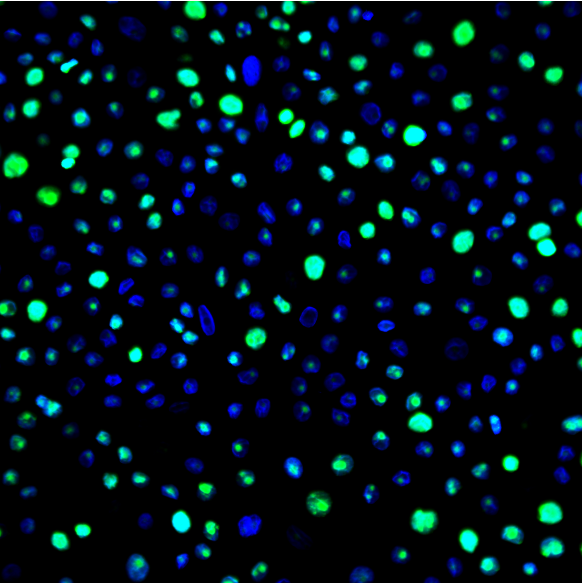

Supplement: Supplementary file 12 — Source data Fig. 2 [file 44321_2026_417_MOESM12_ESM.zip › Figure 2/2M/SiPTENVDR.png]

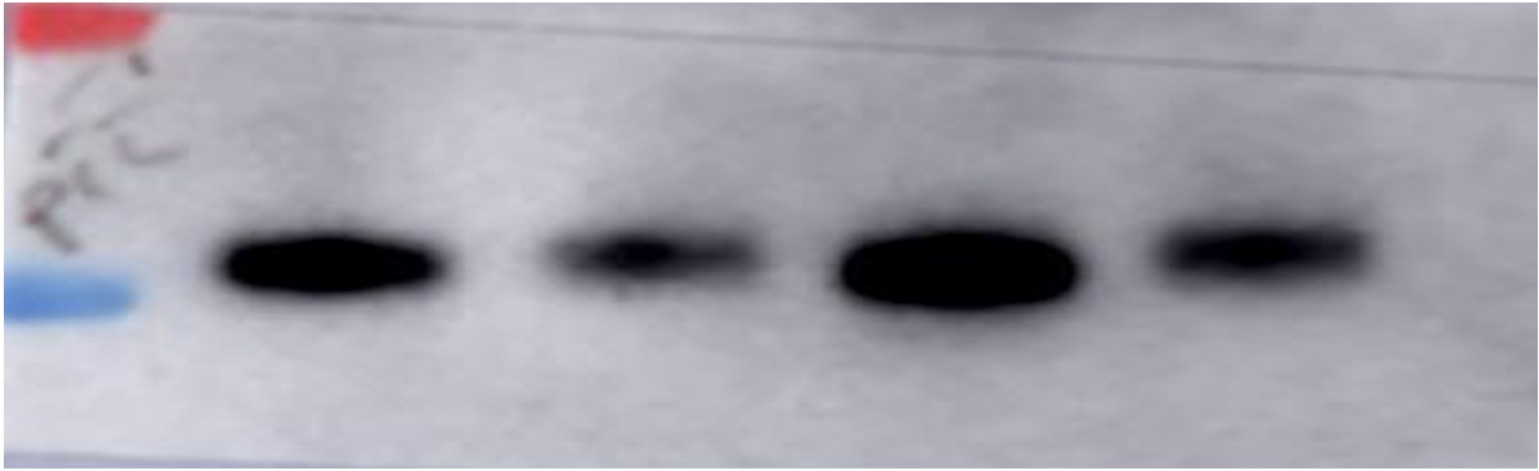

Supplement: Supplementary file 12 — Source data Fig. 2 [file 44321_2026_417_MOESM12_ESM.zip › Figure 2/2K/PTEN.jpg]

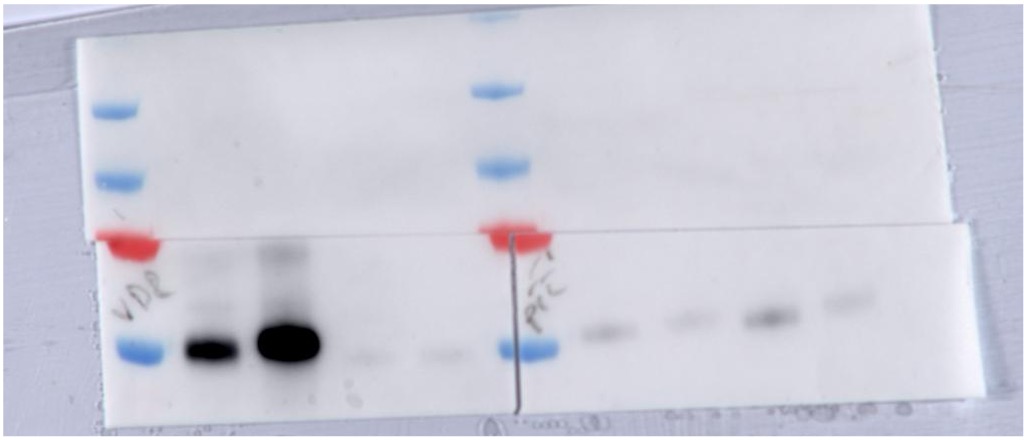

Supplement: Supplementary file 12 — Source data Fig. 2 [file 44321_2026_417_MOESM12_ESM.zip › Figure 2/2K/VDRuncropped.jpg]

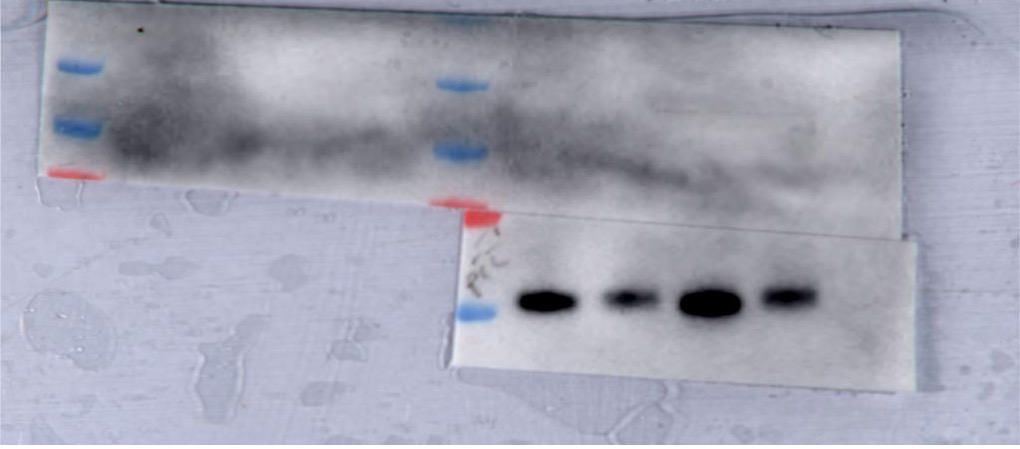

Supplement: Supplementary file 12 — Source data Fig. 2 [file 44321_2026_417_MOESM12_ESM.zip › Figure 2/2K/PTENuncropped.jpg]

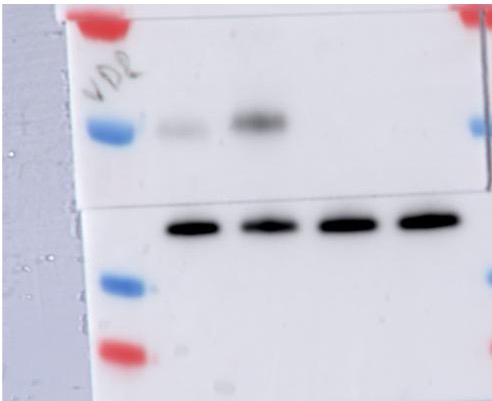

Supplement: Supplementary file 12 — Source data Fig. 2 [file 44321_2026_417_MOESM12_ESM.zip › Figure 2/2K/GAPDHuncropped.jpg]

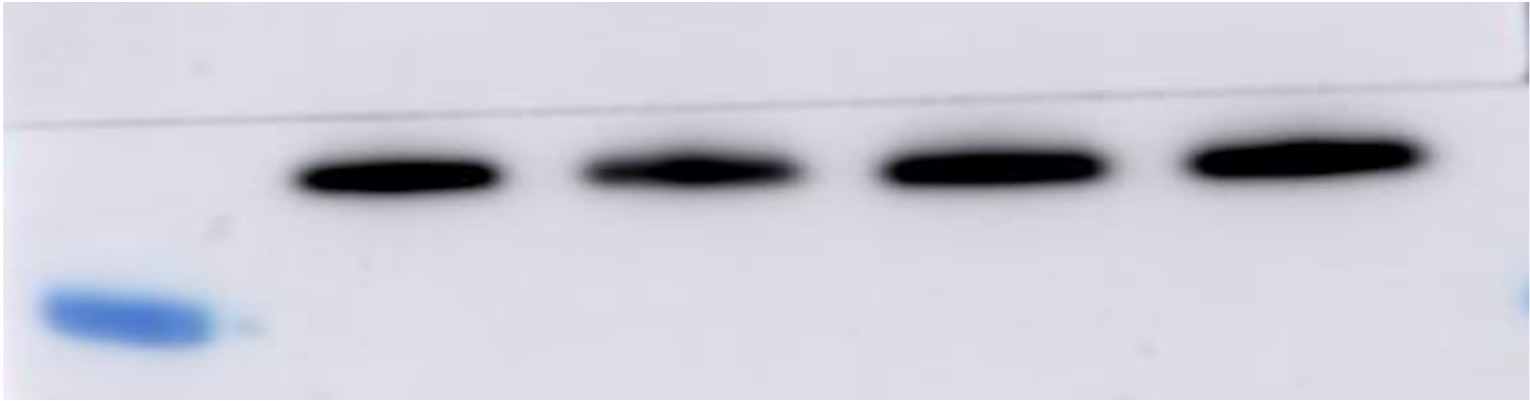

Supplement: Supplementary file 12 — Source data Fig. 2 [file 44321_2026_417_MOESM12_ESM.zip › Figure 2/2K/GAPDH.jpg]

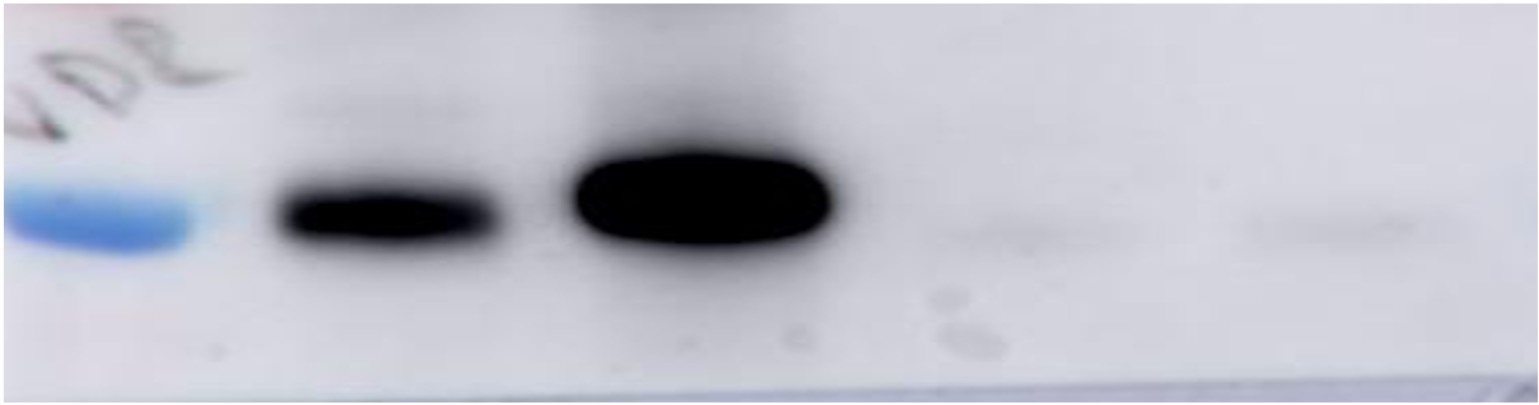

Supplement: Supplementary file 12 — Source data Fig. 2 [file 44321_2026_417_MOESM12_ESM.zip › Figure 2/2K/VDR.jpg]

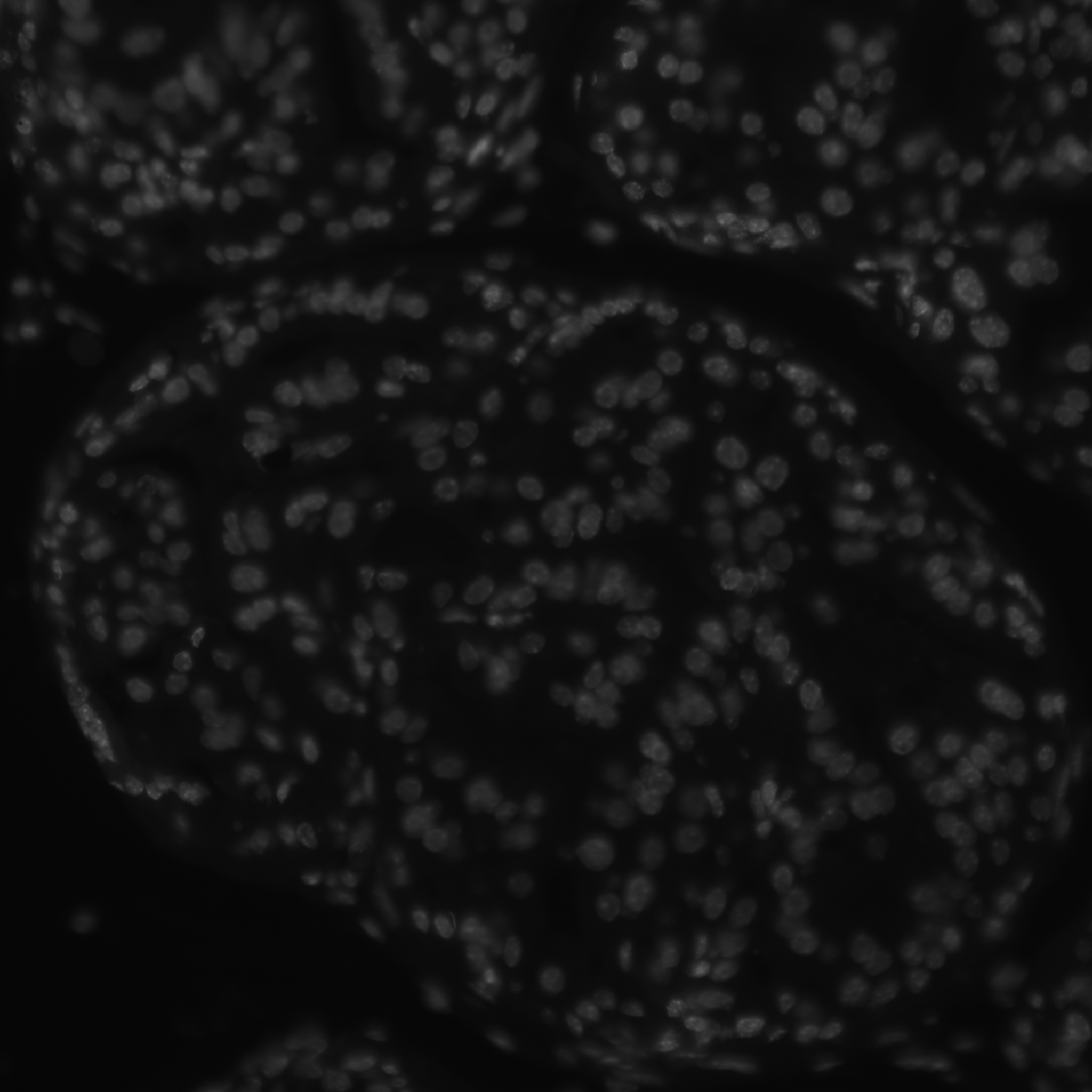

Supplement: Supplementary file 13 — Source data Fig. 3 [file 44321_2026_417_MOESM13_ESM.zip › Figure 3/3K/PTENVDR.tif]

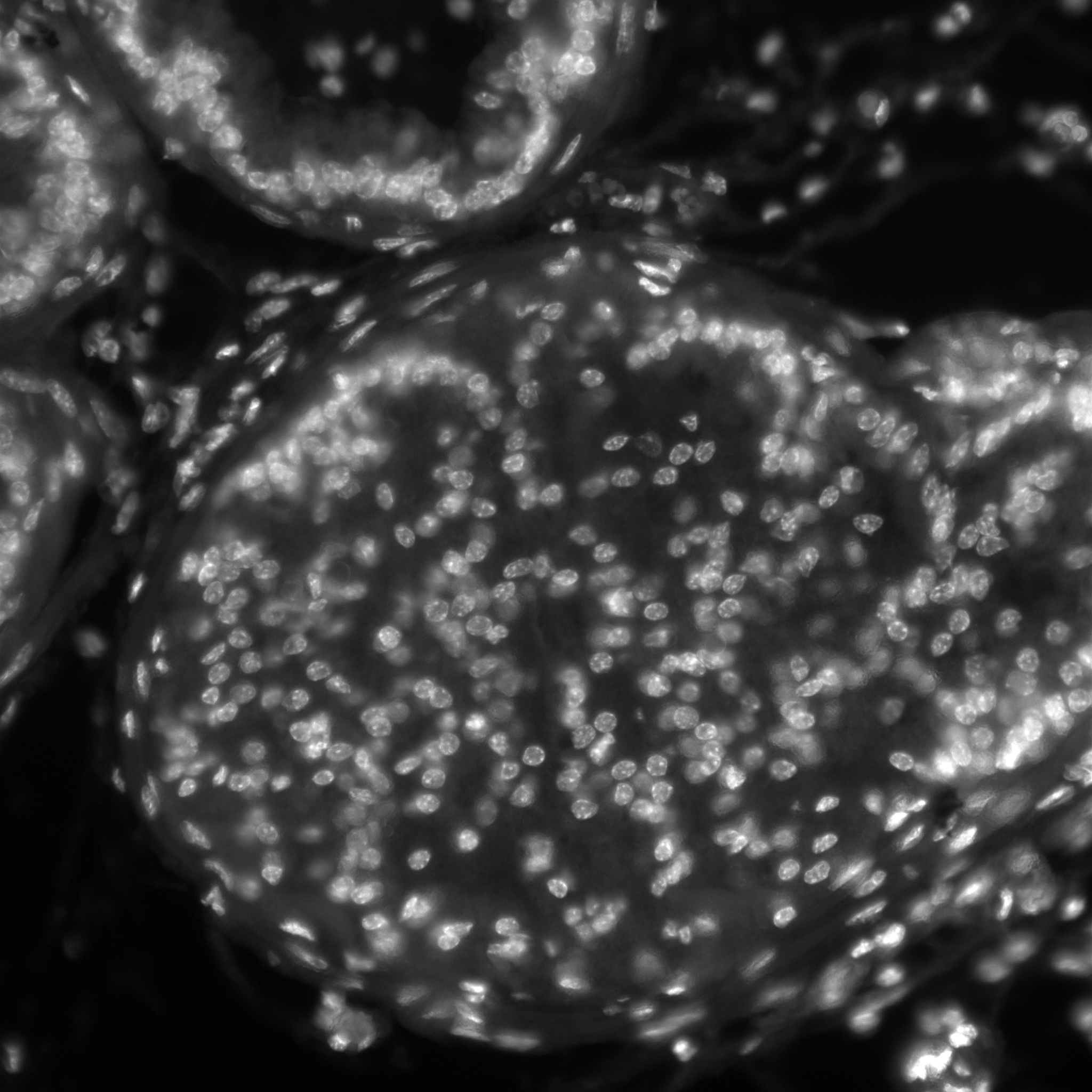

Supplement: Supplementary file 13 — Source data Fig. 3 [file 44321_2026_417_MOESM13_ESM.zip › Figure 3/3K/PTEN.tif]

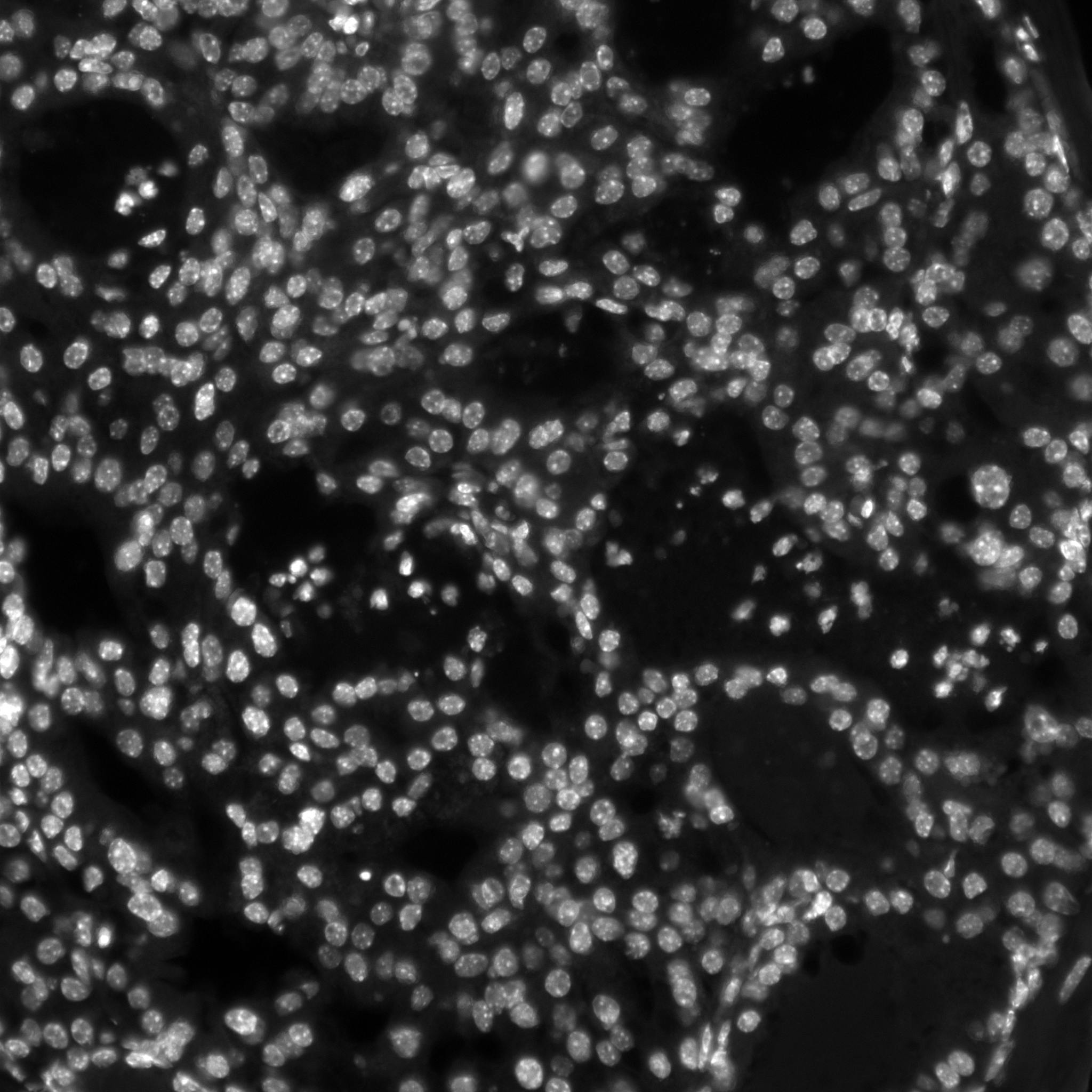

Supplement: Supplementary file 13 — Source data Fig. 3 [file 44321_2026_417_MOESM13_ESM.zip › Figure 3/3L/PTENVDR.tif]

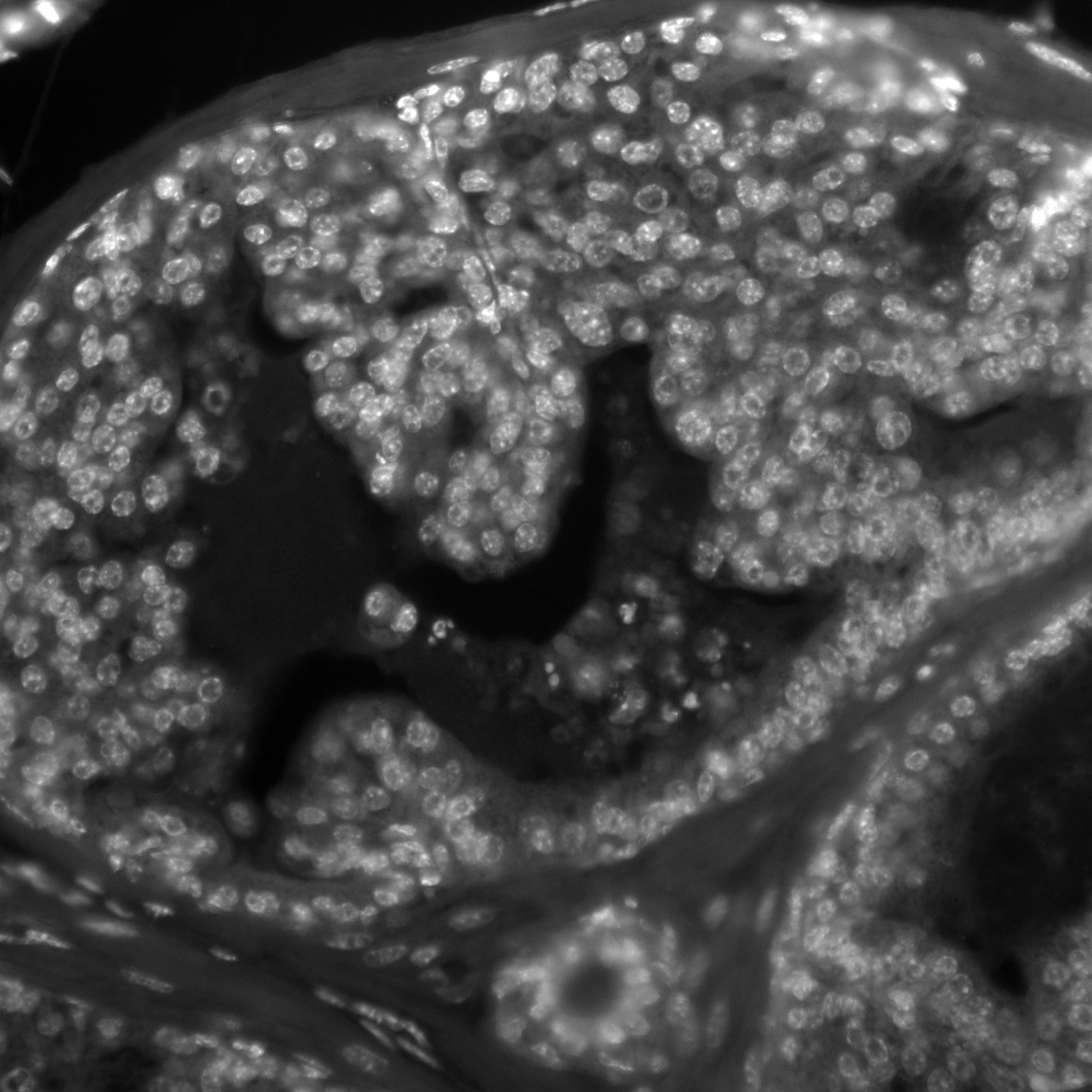

Supplement: Supplementary file 13 — Source data Fig. 3 [file 44321_2026_417_MOESM13_ESM.zip › Figure 3/3L/PTEN.tif]

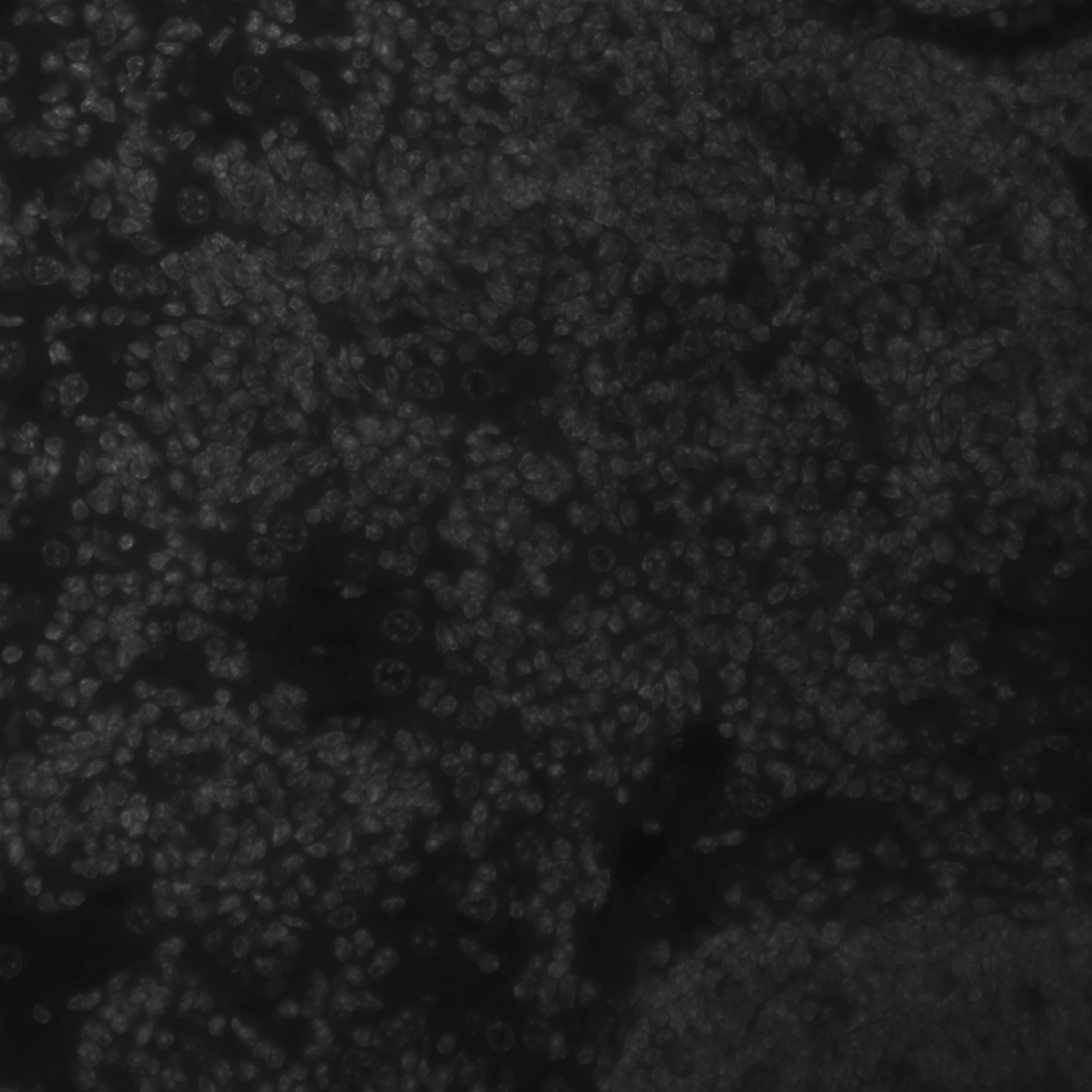

Supplement: Supplementary file 15 — Source data Fig. 5 [file 44321_2026_417_MOESM15_ESM.zip › Figure 5/5O/PTENVDR_vehicle.tif]

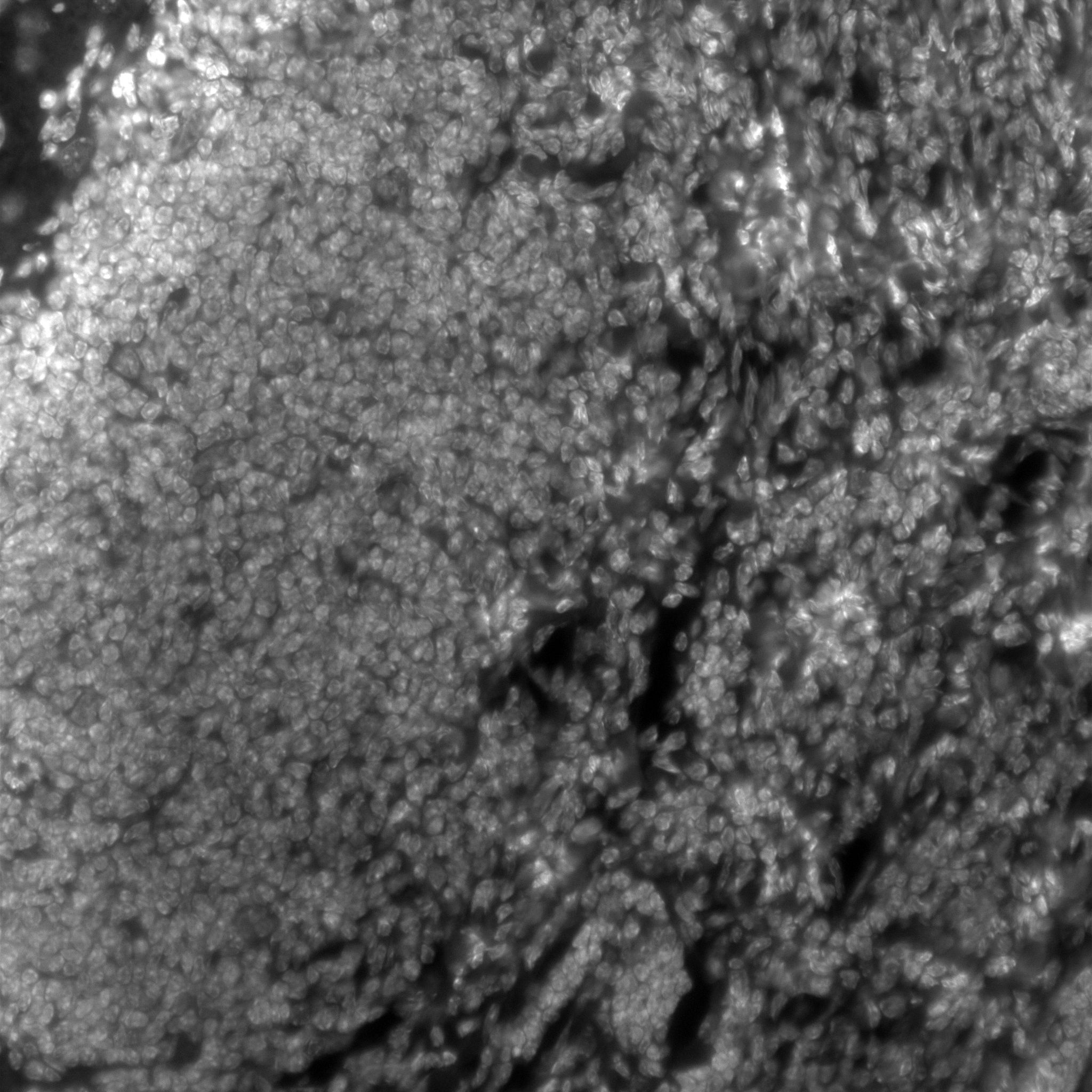

Supplement: Supplementary file 15 — Source data Fig. 5 [file 44321_2026_417_MOESM15_ESM.zip › Figure 5/5O/PTENVDR_SX682.tif]
