# Supplementary material for: Impaired vitamin D signaling reveals neutrophils as key drivers of prostate cancer dissemination
Source: EMBO Mol Med. 2026 Apr 10;18(5):1967–89. doi: 10.1038/s44321-026-00417-5 (PMC13179334; doi:10.1038/s44321-026-00417-5)
Supplement: Supplementary file 16 — Expanded View Figures [file 44321_2026_417_MOESM16_ESM.pdf]

Expanded View Figures

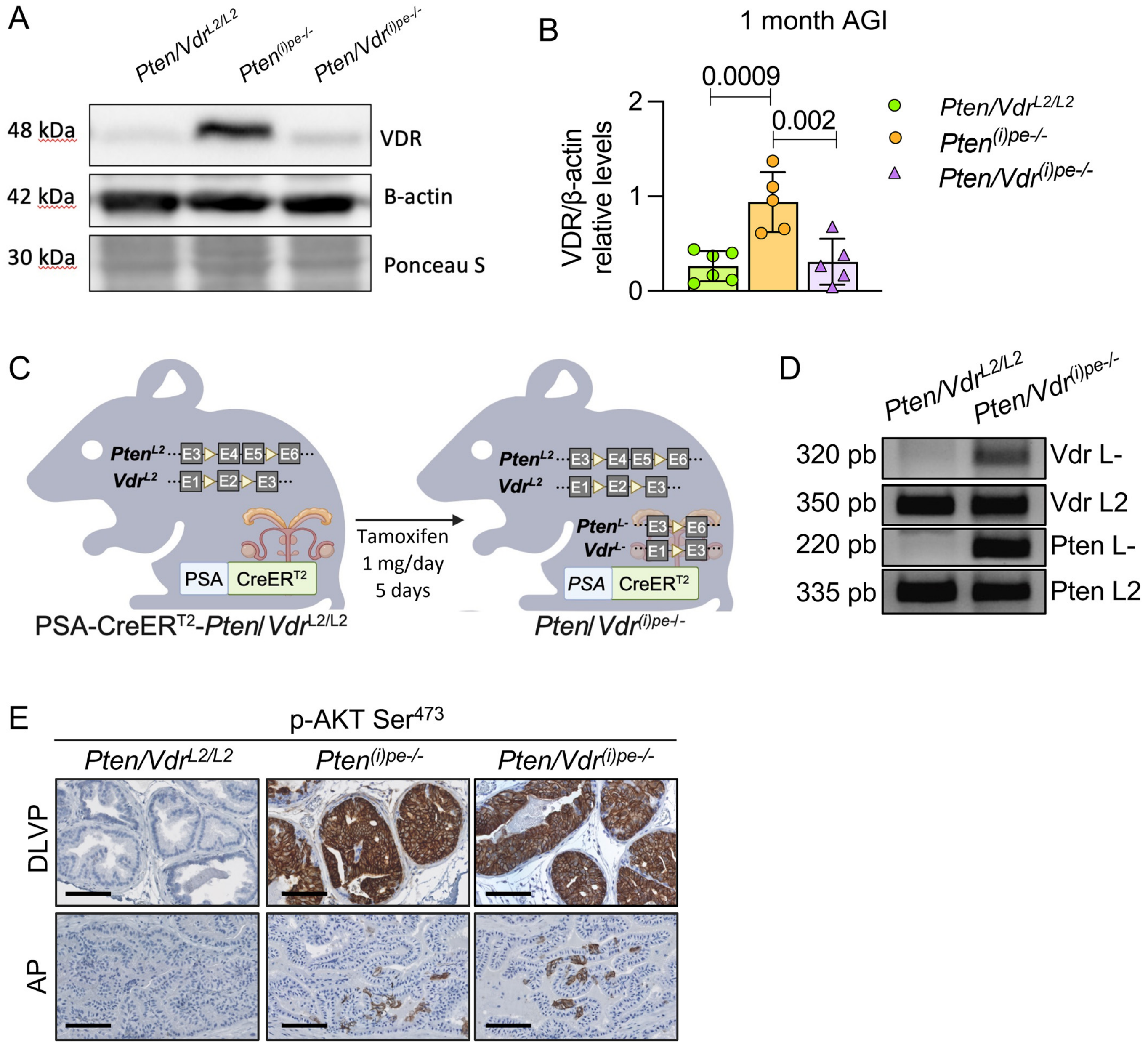

Figure EV1. Generation and characterization of *Pten/Vdr*$^{(i)pe-/-}$ mice.

Representative VDR immunoblot (**A**) and relative expression (**B**) determined in prostatic protein extracts from *Pten/Vdr*$^{L2/L2}$, *Pten*$^{(i)pe-/-}$ and *Pten/Vdr*$^{(i)pe-/-}$ mice 1 month AGI. β-actin was used as a loading control. $n = 5$–6 mice/group. Data are represented as mean +/− standard deviation. *p* value determined by two-way ANOVA with Tukey's post-hoc is indicated. (**C**) Schematic representation of the generation of *Pten/Vdr*$^{(i)pe-/-}$ mice. Yellow triangle represents LoxP sites. pe, prostatic epithelium; i, inducible; PSA, prostatic specific antigen; L2, floxed allele; L-, excised allele. (**D**) Representative image of the PCR products of the *Vdr* L-, *Pten* L-, *Vdr* L2 and *Pten* L2 alleles in DNA extracts from prostates of *Pten/Vdr*$^{L2/L2}$ and *Pten/Vdr*$^{(i)pe-/-}$ mice 1 month AGI. (**E**) Representative p-Akt Ser^{473} immunostaining on DLVP and AP sections from *Pten/Vdr*$^{L2/L2}$, *Pten*$^{(i)pe-/-}$ and *Pten/Vdr*$^{(i)pe-/-}$ mice, 1 month AGI. Scale bar = 250 µm. $n = 4$ mice/group.

© The Author(s)

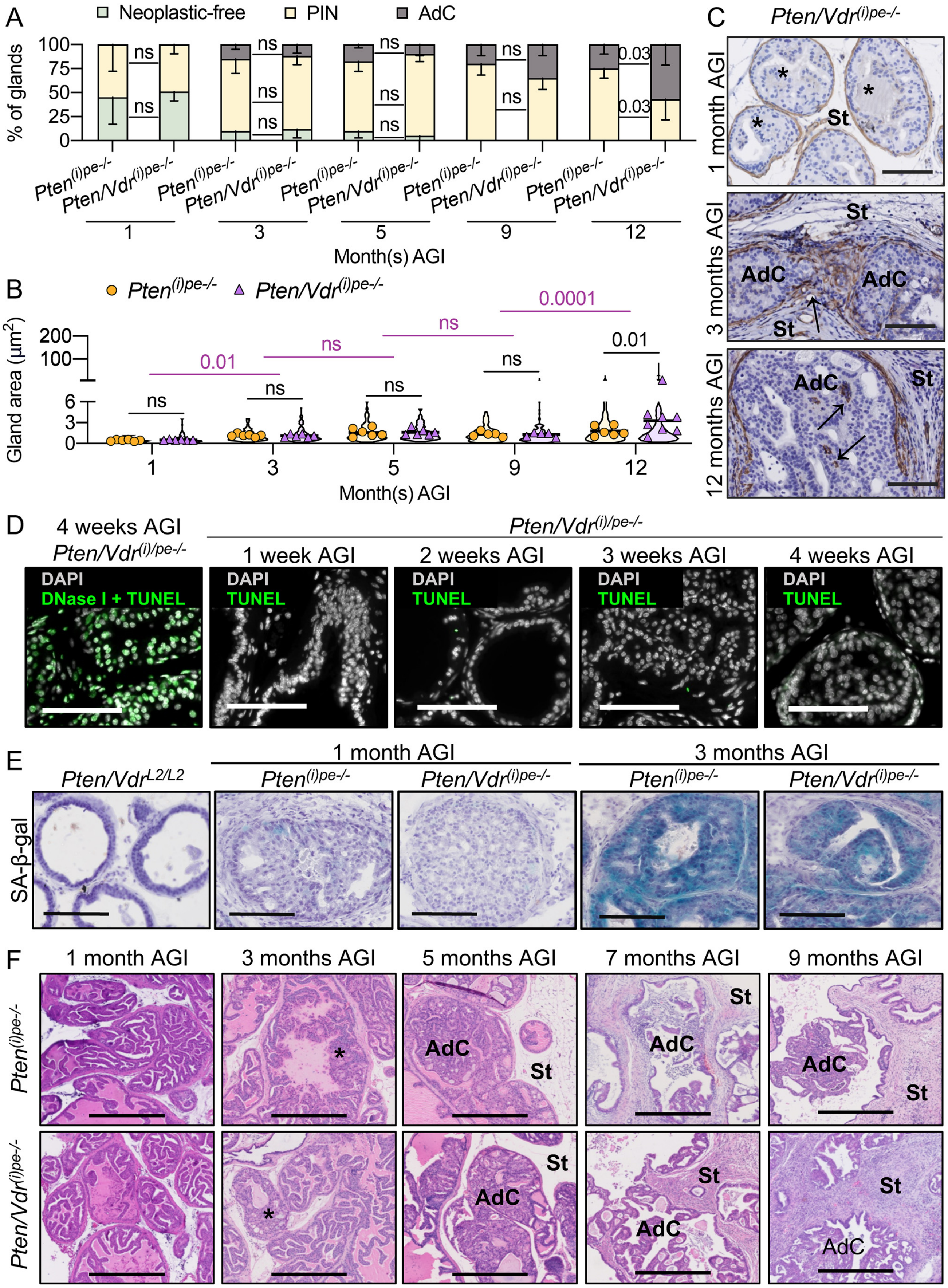
A
Neoplastic-free
PIN
AdC
% of glands
ns
0.03
Month(s) AGI
B
Gland area (μm²)
0.01
ns
0.0001
Month(s) AGI
C
1 month AGI
3 months AGI
12 months AGI
St
AdC
D
4 weeks AGI
1 week AGI
2 weeks AGI
3 weeks AGI
DAPI
DNase I + TUNEL
TUNEL
E
1 month AGI
3 months AGI
SA-β-gal
F
1 month AGI
3 months AGI
5 months AGI
7 months AGI
9 months AGI

Figure EV2. Histological evaluation of prostatic tumors in *Pten*$^{(i)pe-/-}$ and *Pten/Vdr*$^{(i)pe-/-}$ mice.

(**A**) Histological scoring of DLVPs from *Pten*$^{(i)pe-/-}$ and *Pten/Vdr*$^{(i)pe-/-}$ mice analyzed at 1, 3, 5, 9, and 12 months AGI. AdC, adenocarcinoma. $n = 5$ mice/group. Data are represented as mean - standard deviation. p values determined by two-way ANOVA with Tukey's post-hoc are indicated. ns, $p \geq 0.05$. (**B**) Gland area quantification on DLVP sections from *Pten*$^{(i)pe-/-}$ and *Pten/Vdr*$^{(i)pe-/-}$ mice 1, 3, 5, 9, and 12 months AGI. $n = 7$ mice/group. p values determined by two-way ANOVA with Tukey's post-hoc are indicated. ns, $p \geq 0.05$. (**C**) Representative α-SMA immunostaining on DLVP sections from *Pten/Vdr*$^{(i)pe-/-}$ mice 1, 3, and 12 months AGI. Scale bar = 100 µm. $n = 3$ mice. *, PIN; →, invasion/adenocarcinoma; AdC, adenocarcinoma; St, stroma. (**D**) Representative images of TUNEL assay performed on DLVP sections from *Pten/Vdr*$^{(i)pe-/-}$ mice 1-, 2-, 3-, and 4-week(s) AGI. DNAse I treated DLVP section was used as a positive control. TUNEL/fluorescein (green) and DAPI (gray). Scale bar = 100 µm. $n = 2$ mice/group. (**E**) Representative SA-β-galactosidase staining of DLVP sections from *Pten/Vdr*$^{L2/L2}$, *Pten*$^{(i)pe-/-}$ and *Pten/Vdr*$^{(i)pe-/-}$ mice 1 and 3 months AGI. $n = 3$ mice/group. Scale bar = 250 µm. (**F**) Representative HE staining of AP sections from *Pten*$^{(i)pe-/-}$ and *Pten/Vdr*$^{(i)pe-/-}$ mice 1, 3, 5, 7, and 9 months AGI. Scale bar = 250 µm. $n = 5$ mice/group. *, PIN; AdC, adenocarcinoma; St, stroma.

© The Author(s)

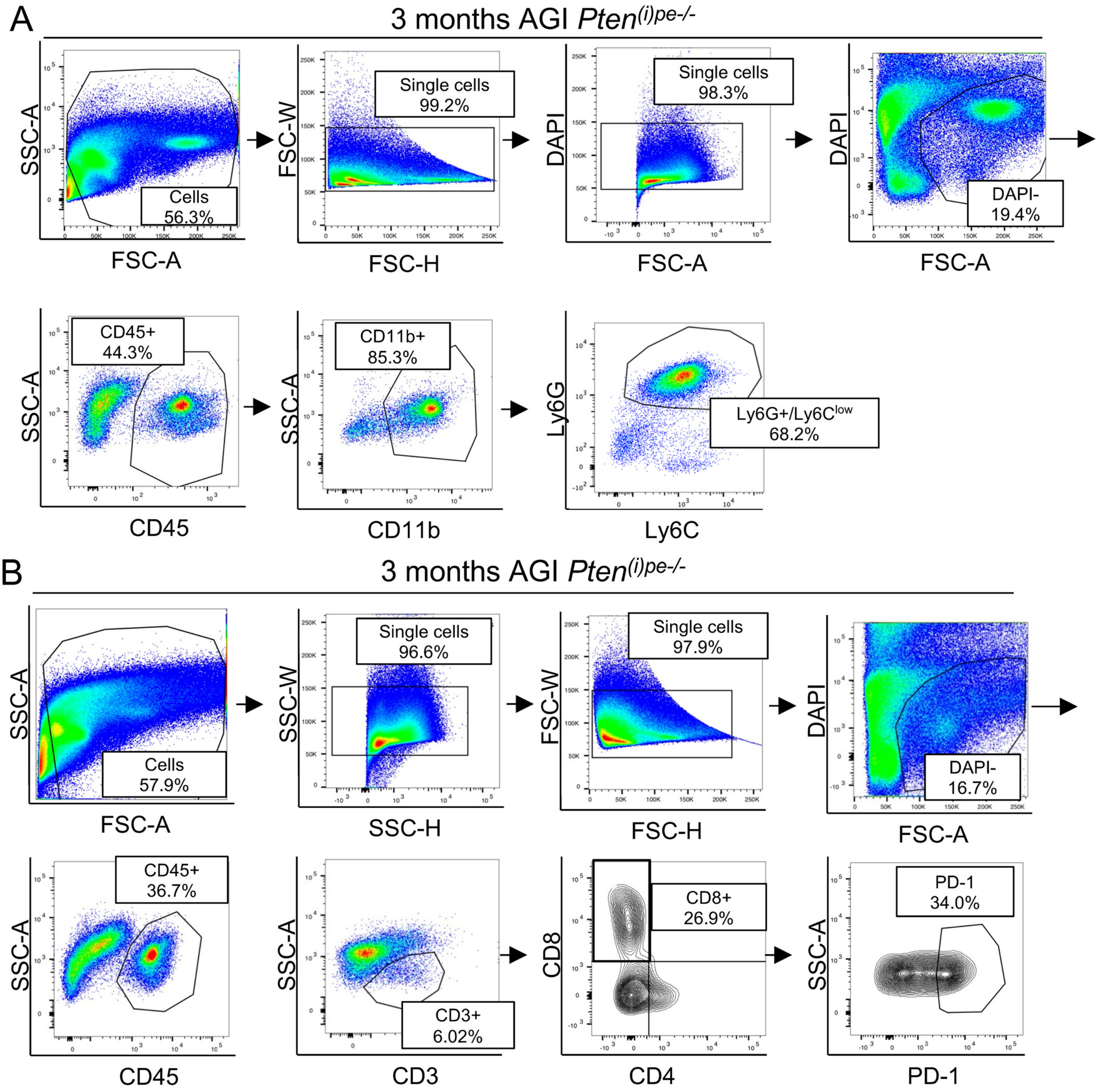

Figure EV3. Flow cytometry analysis of neutrophils and T cells.

Representative gates for flow cytometry analysis of neutrophils (**A**) and T cells (**B**) in dissociated DLVPs from *Pten*$^{(i)pe-/-}$ mice 3 months AGI. $n > 5$.

 © The Author(s)

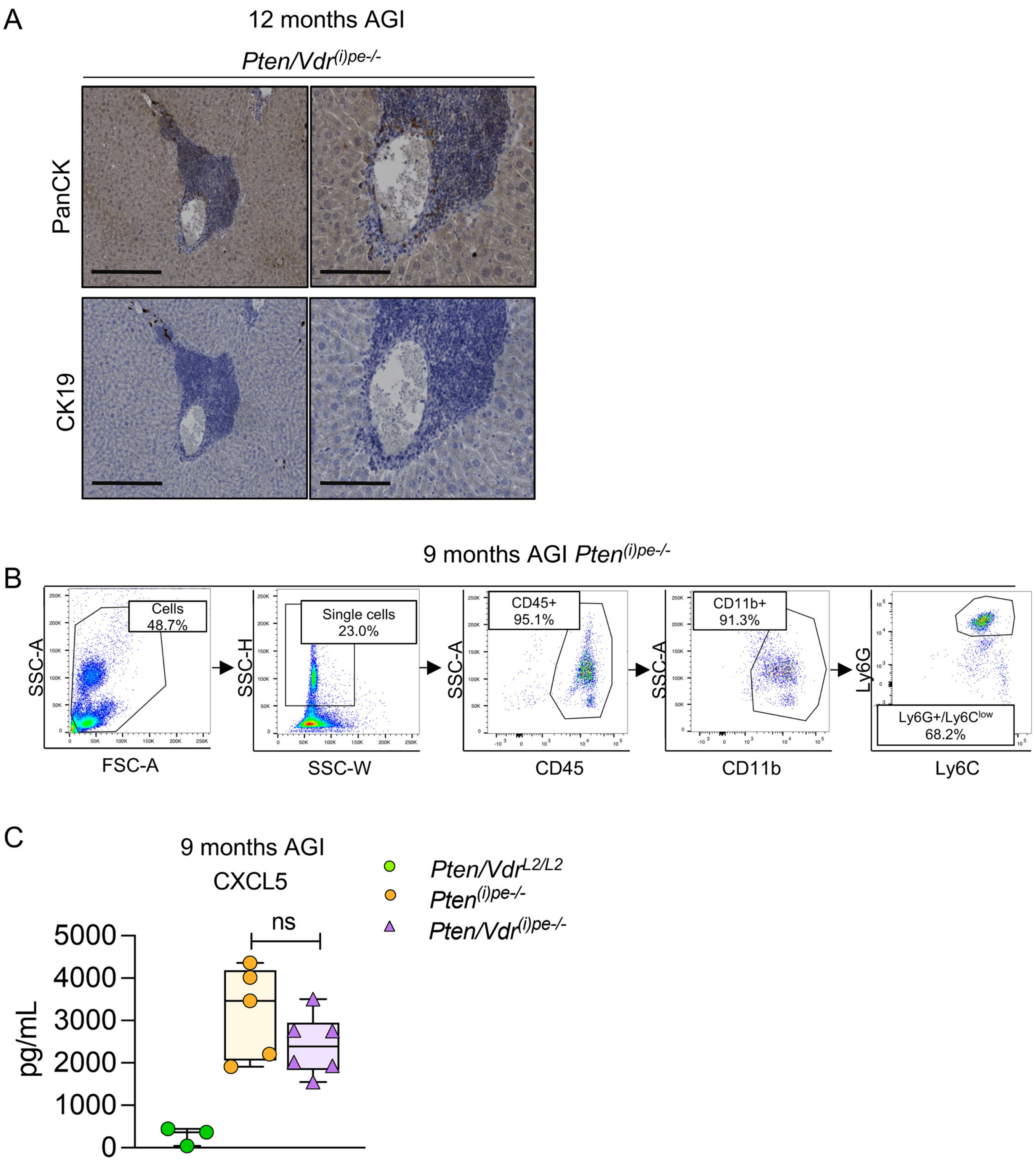

Figure EV4. Characterization of liver metastasis and neutrophils infiltration.

(**A**) Representative immunostaining of PanCK and CK19 on liver sections from *Pten/Vdr*$^{(i)pe-/-}$ mice 12 months AGI. Scale bar 250 µm (large view) and 50 µm (zoom). $n = 3$ mice. (**B**) Representative gates for flow cytometry analysis of neutrophils in blood from *Pten*$^{(i)pe-/-}$ mice 9 months AGI. $n = 8$. (**C**) CXCL5 levels determined in protein extracts from DLVPs of *Pten/Vdr*$^{L2/L2}$, *Pten*$^{(i)pe-/-}$ and *Pten/Vdr*$^{(i)pe-/-}$ mice 9 months AGI. $n = 3$ *Pten/Vdr*$^{L2/L2}$, $n = 5$ *Pten*$^{(i)pe-/-}$ and $n = 6$ *Pten/Vdr*$^{(i)pe-/-}$ mice. The boxes extend from the 25th to 75th percentiles, the lines represent the median, and the whiskers go down to the smallest up to the largest value. ns: not significant.
