# Supplementary material for: Impaired vitamin D signaling reveals neutrophils as key drivers of prostate cancer dissemination
Source: EMBO Mol Med. 2026 Apr 10;18(5):1967–89. doi: 10.1038/s44321-026-00417-5 (PMC13179334; doi:10.1038/s44321-026-00417-5)
Supplement: Supplementary file 13 — Source data Fig. 3 [file 44321_2026_417_MOESM13_ESM.zip › Figure 3/3C/Table EV5.zip!/Table_EV5.docx]

**Table EV5 :** Output file from the ClusterProfiler analysis using differentially expressed genes from the single cell analysis from *Pten/Vdr^(i)pe-/-^* and *Pten^(i)pe-/-^* mice in each cluster (Table_EV4). ID and Description correspond to the name of the KEGG pathway. GeneRatio is the number of gene within the pathway / total number of gene in this pathway. BgRatio : number of gene in this pathway / total number of gene in the dataset used. Pvalue and qvalue, are the statistical values of the gene set enrichment analysis. geneID depicts the differentially expressed genes within the corresponding pathway. Count is the number of gene associated with this pathway in the dataset.
